# Molecular and Clinical Insights in Malignant Brenner Tumor of the Testis With Liver Metastases:A Case Report

**DOI:** 10.3389/fonc.2021.663489

**Published:** 2021-04-12

**Authors:** Pietro Parcesepe, Luigi Coppola, Andrea Remo, Mario Rosario D’Andrea, Giulia Coppola, Michele Simbolo, Erminia Manfrin, Aldo Scarpa, Elena De Santis, Guido Giordano

**Affiliations:** ^1^ Section of Pathology, Department of Diagnostics and Public Health, University and Hospital Trust of Verona, Verona, Italy; ^2^ Unità Operativa Complessa (UOC) Anatomia ed Istologia Patologica e Citologia Diagnostica, Dipartimento dei Servizi Diagnostici e della Farmaceutica, Ospedale Sandro Pertini, Roma, Italy; ^3^ Pathology Unit, “Mater Salutis” Hospital, Legnago, Italy; ^4^ Unità Operativa Semplice a valenza Dipartimentale (UOSD) Oncologia, Ospedale S. Paolo, Civitavecchia, Italy; ^5^ Department of Radiological, Oncological and Pathological Sciences, Sapienza University of Rome, Rome, Italy; ^6^ ARC-Net Centre for Applied Research on Cancer, University and Hospital Trust of Verona, Verona, Italy; ^7^ Department of Anatomical, Histological, Forensic Medicine and Orthopedic Sciences, Sapienza University of Rome, Rome, Italy; ^8^ Unit of Medical Oncology and Biomolecular Therapy, Polilinico Riuniti, Foggia, Department of Medical and Surgical Sciences, University of Foggia, Foggia, Italy

**Keywords:** Brenner tumor, testis, epididymis, FGFR, malignant, mutations, case report, liver metastases

## Abstract

Malignant Brenner Tumor (mBT) is extremely rare. Although BT are almost exclusive ovarian neoplasms, they may constitute a highly unusual tumor of the testis; in fact, only seven fully documented cases have been reported to date. Because of their rarity, the pathogenesis of these tumors has not been clarified and there is no standard therapeutic approach. We report the first case of epididymal mBT with synchronous, multiple, liver metastases and a very dramatic clinical course. Both primary tumor and metastasis were subjected to mutational analysis of 20 cancer associated genes. Primary tumor showed *FGFR3* Tyr375Cys and *PIK3CA* His1047Arg missense mutations. Both mutations are reported as pathogenic in ClinVar database. The same *FGFR3* mutation was present in liver metastasis. Based on these results we believe that the FGFR pathway could be an ideal candidate for personalized treatment, offering hope to a subset of patients with mBT. Personalized approach, including mutational analysis and molecular testing should be required in patients with rare tumors in order to clarify diagnosis and improve therapeutic strategies.

## Introduction

Brenner tumor (BT) is an uncommon subtype of ovarian neoplasm included within the surface epithelial-stromal tumors. It was first described by Brenner in 1907 and accounts for ~1.4-2.5% of all ovarian tumors (1, 2). BT are classified into three categories according to the World Health Organization (WHO), namely benign, borderline and malignant (3). While the vast majority of BTs are benign, around 2-5% are borderline and malignant variants (4). Extra-ovarian BT are extremely rare, and are mainly found in the broad ligament, uterus, vagina, testis and epididymis (5). To the best of our knowledge, only 7 cases of testicular BT have been reported to date (5–11). Ovarian tumors are divided into two broad categories named type I and type II. Type I tumors include low-grade serous carcinoma, mucinous carcinoma, endometrioid carcinoma, malignant BT (mBT), and clear cell carcinoma. The tumorigenic pathway for type I tumors is characterized by clearly recognized precursor lesions, namely, cystadenoma, atypical proliferative tumor, and noninvasive carcinoma. Traditionally, the latter two non-invasive tumors have been combined into one category designated as “borderline.” Type II tumors are currently classified as moderately and poorly differentiated serous carcinoma (high-grade serous carcinoma), malignant mixed mesodermal tumors (carcinosarcomas), and undifferentiated carcinoma. Notably, type I tumors evolve slowly and are associated with distinct molecular changes that are rarely found in type II tumors, such as *BRAF* and *KRAS* mutations for serous tumors, *KRAS* mutations for mucinous tumors, *CTNNB1* and *PTEN* mutations for endometrioid tumors. In contrast, type II tumors evolve rapidly and metastasize early in their course. There is very limited data on the molecular alterations associated with type II tumors except for frequent mutations of *TP53* in high-grade serous carcinomas and malignant mixed mesodermal tumors (carcinosarcomas) (3, 12). Due to their rare occurrence, there is lack of information about the molecular features of extraovarian mBT and there is no standard therapeutic approach. We report here a case of epididymal mBT with synchronous liver metastases. Both primary tumor and metastasis underwent mutational analysis of 20 cancer genes.

## Materials and Methods

Patient’s medical history including comorbidities, concomitant medications, BT diagnosis and treatment were taken from clinical records. Written informed consent for the case publication was obtained from the patient. Three-micrometer-thick sections were cut from formalin fixed paraffin-embedded tissues for immunohistochemistry (IHC) and routinely stained with hematoxylin and eosin (H&E) (13). IHC analysis was performed for CKAE1/AE3, CK7 CK5, P63, CK20, EMA, Vimentine, Calretinine, TTF1 and PSA. Four 10-micrometer paraffin sections were used for gene profile assessment. The different tumor areas were manually micro-dissected to ensure that each tumor sample contained at least 80% neoplastic cells. Normal testicular tissue was used as a control. DNA was isolated with the QIAmp DNA FFPE tissue kit (Qiagen, Milan, Italy) and quantitated using the Qubit 2.0 fluorometer (Life Technologies, Milan, Italy). DNA (20 ng) was used for multiplex polymerase chain reaction amplification, using the Ion AmpliSeq Cancer Panel (Life Technologies, Milan, Italy) which explore the following 20 cancer associated genes: *APC, ATM, BRAF, CDH1, CDKN2A, CTNNB1, EGFR, ERBB2, ERBB4, FBXW7, FGFR3, FLT3, GNAS, HRAS, KRAS, NRAS, KDR, PIK3CA, SMAD4, TP53.* To assess the significance of gene mutations, SIFT, PolyPhen and ClinSig bio-informatics tools were interrogated, in order to predict possible impact of an amino-acid substitution on the structure and function of human proteins. Ethic Committee approved all procedures.

## Results

### Case Presentation

**Figure 1:**
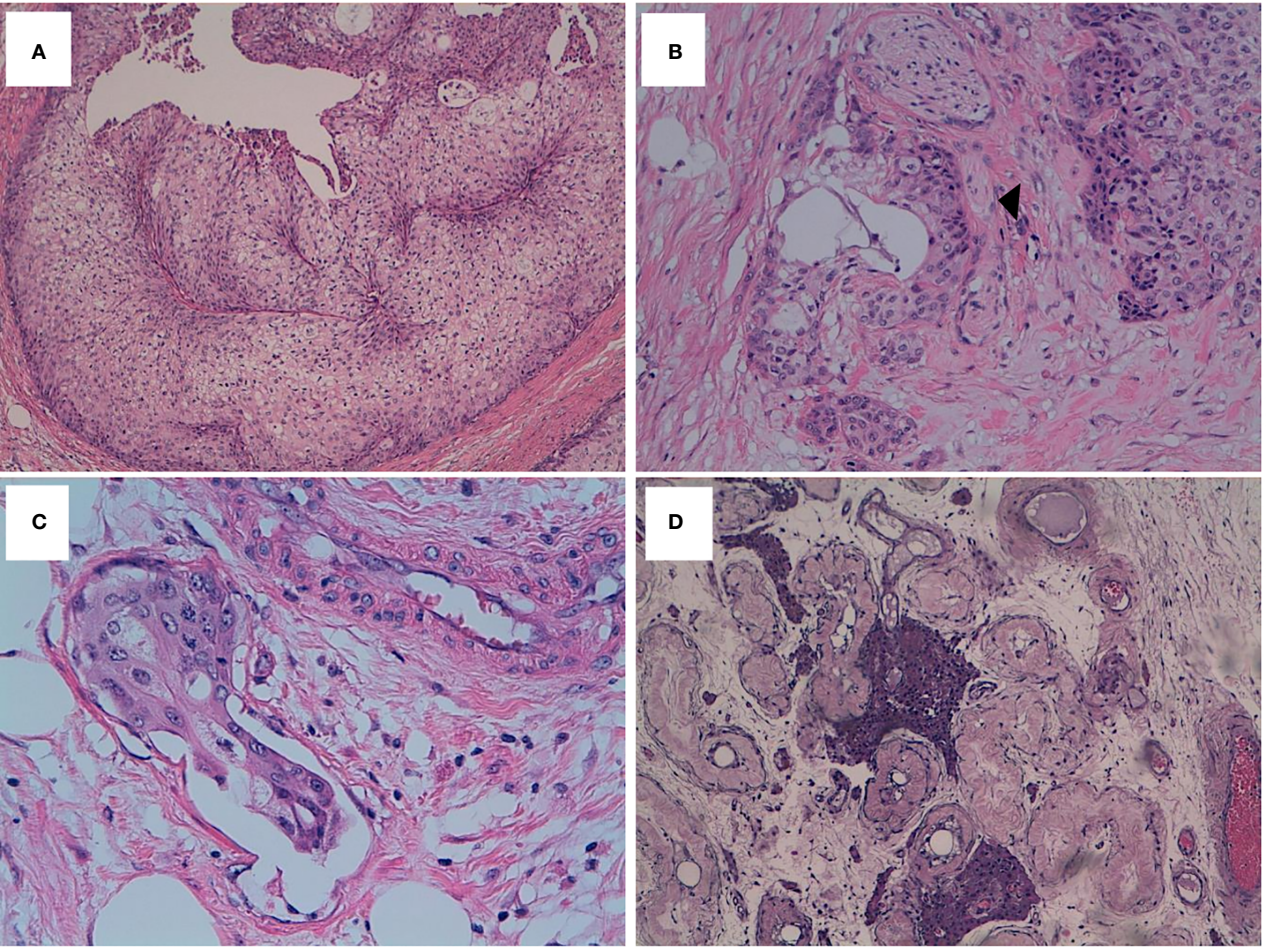
mBT tissue stained with H&E. **(A)** Neoplatic cells resembling invasive urothelial carcinoma exhibit high mitotic activity (H&E, x10 magnification). **(B)** Perineural (*head arrow*) infiltration (x20 magnification). **(C)** Linfovascular invasion (x40 magnification). **(D)** Testis showing tubular atrophy and Leydig cells hyperplasia (x10 magnification).

On October 2015, a 78-years old male presented left testicular mass with swelling. His family Doctor suggested testicular ultrasound that showed a dishomogeneous left testicular mass measuring around 2 cm. Blood examination evidenced elevated liver enzymes and mild renal impairment. Contrast-enhanced computed tomography (CT) of chest and abdomen was performed and multiple liver metastases, left adrenal and kidney lesion, renal- and superior- cava vein thrombosis were detected. After surgical evaluation, the patient underwent to left orchiectomy for testicular mass. Histological examination and immunohistochemistry showed morphological features of malignant BT of epididymis (**Figures 1A, B**). At the time of hospitalization, patient had Karnofsky Performance Status (KPS) of 70%. Laboratory examinations confirmed mild renal impairment (serum creatinine = 1.78 mg/dl, uricemia = 8.1 mg/dl, blood urea nitrogen = 70 mg/dl); elevated liver enzymes (Aspartate aminotransferase = 110 U/l, alanine aminotransferase = 74 U/l, gamma-glutamyl transferase = 241 U/l); grade 1 anemia (hemoglobin = 9.6 gr/dl). Tumor markers for testicular cancer, such as α-fetoprotein and β-HCG were negative, as well as LDH was within normal values. To exclude a second tumor, on November 2015, the patient was admitted in Oncology division and liver biopsy was performed. Histological examination confirmed mBT metastasis. Due to the worsening of clinical conditions (KPS 50%), no chemotherapy was indicated and best supportive care was started. Unfortunately, because of metastatic worsening disease the patient died four months after the diagnosis.

### Histological Diagnosis

On gross pathological examination, the resected specimen was composed by testis sized cm 2 × 1.5 × 1.5 with hemorrhagic features of tunica vaginalis and cm 5 of spermatic cord. The cut surface showed a tan-grey solid-nodular neoplasm of cm 2,5 diameter infiltrating the epididymis and the spermatic cord. The histological evaluation revealed an epithelial neoplastic proliferation composed by large nests of transitional epithelium surrounded by septa of dense fibrous tissue. Some nests presented central cavities containing necrotic material. Most of the neoplastic cells showed round to oval nuclei with evident nucleoli and abundant eosinophilic cytoplasm. Single scattered cells showed a pleomorphic and voluminous nucleus. Perineural and linfovascular invasion were observed (**Figure 1C**). The tumor displayed high mitotic activity with evidence of atypical mitosis. The proliferative index of the neoplastic cells evaluated with Ki67 was 40%.

**Figure 2:**
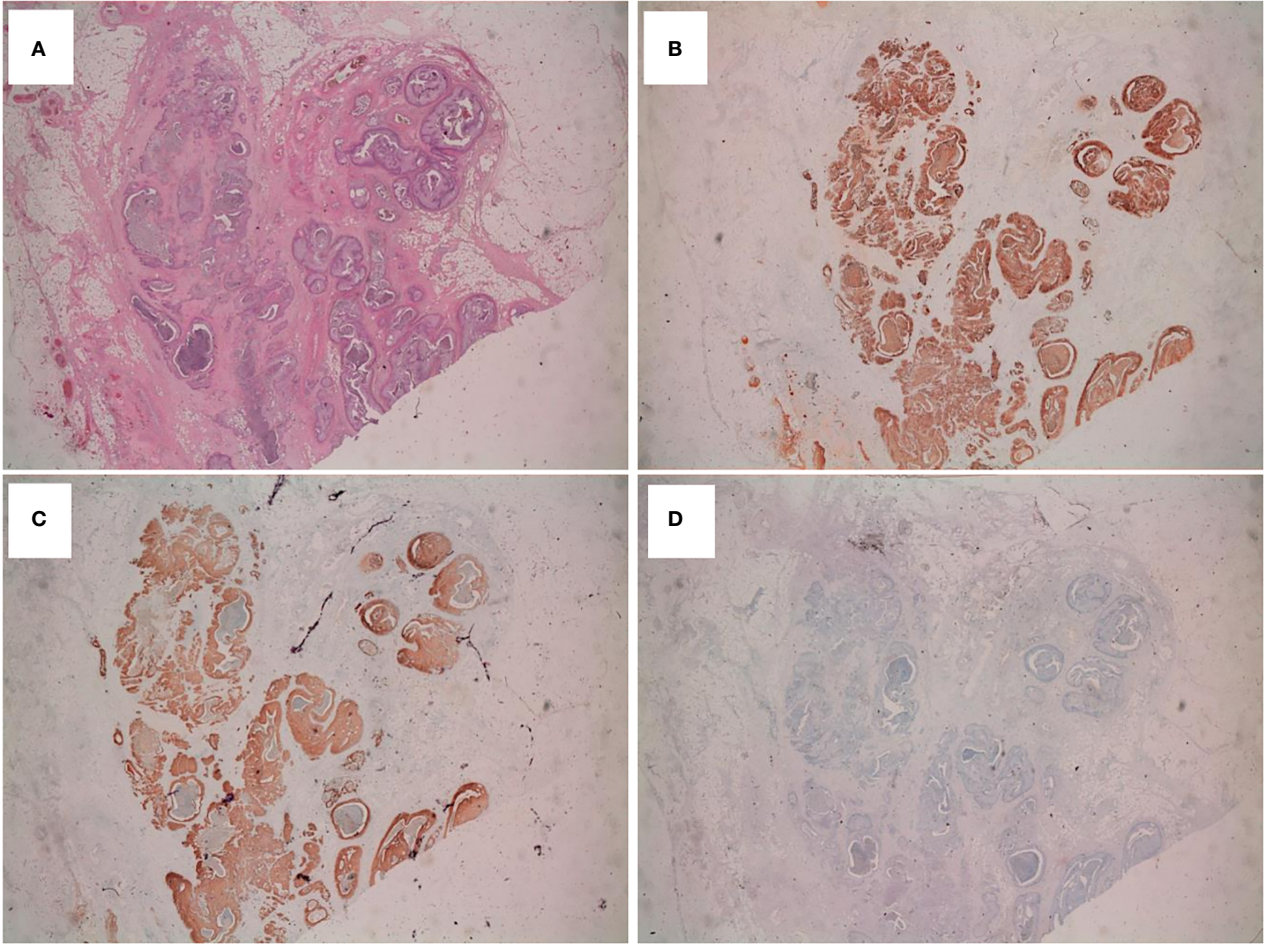
Immunohistochemical analysis on mBT tissue sections **(A)** Testicular lesion showing several dilated cystic-like areas (H&E; x20 magnification). Immunohistochemical staining of tumor tissue showing intense and diffuse immunopositivity for CK7 (x20) **(B)** and EMA (x20) **(C)**. Tumor tissue was CK20 negative (x20) **(D)**.

The immunohistochemical analysis revealed an immunophenotype compatible with mBT: CKAE1/AE3 positive, CK7 positive, EMA positive, P63 mild positivity, CK5 focal positivity, CK20 negative, Vimentin negative, Calretinin negative, TTF-1 negative, PSA negative (**Figures 2A–D**).

After a few months, the patient developed a metastatic lesion to the liver. The microscopic evaluation of the liver biopsy showed the same morphological and immunohistochemical aspects of the primary lesion. Furthermore, the IHC for CK20, CDx2, TTF1, CD10 and Vimentin showed a negative result, thus excluding metastases from gastrointestinal, lung, thyroid, renal cancer as well as sarcoma, respectively.

### Molecular Findings

Deep sequencing of DNA from paraffin embedded tissues of both primary testicular BT and related liver metastasis identified mutations in *PIK3CA* and *FGFR3*. In particular, the primary tumor showed *FGFR3* Tyr375Cys and *PIK3CA* His1047Arg point (missense) mutations. Both mutations are classified as pathogenic in ClinVar data base (http://www.clinvar.com). The metachronous liver metastasis had the same *FGFR3* mutation but lacked the *PIK3CA* mutation) (**Table 1**).

**Table 1:** Genetic mutations and their possible predictive impact.

Site	Chrom	POS	REF	ALT	Gene	Consequence	HGVSp	Clin sig
Primary	4	1806099	A	G	*FGFR3*	missense_variant	NP_001156685.1:p.Tyr375Cys	P
Primary	3	178952085	A	G	*PIK3CA*	missense_variant	NP_006209.2:p.His1047Arg	P
Metastasis	4	1806099	A	G	*FGFR3*	missense_variant	NP_001156685.1:p.Tyr375Cys	P

Chrom, Chromosome; POS, Position; REF, Reference; Pri, Primary tumor; Met, Metastatic Tumor; A, Adenosine; ALT, Alteration; G, Guanine; HGVSp, Human Genome Variation Society Protein; Tyr, Tyrosine; Cys, Cysteine; His, Histidine; Arg, Arginine; PD, Possible Damaging; P, Pathogenic.

## Discussion

Malignant BT (mBT) is extremely rare, comprising less than 5% of all BT (14). Due to its rarity the pathogenesis of this tumor type remains to be clarified. Initially, origin from ovarian surface epithelium was supposed; however, latter studies indicate possible derivation from Walthard cell nest, within normal ovaries and fallopian tubes (15–17). Interestingly, BTs demonstrate transitional-type differentiation as is seen in bladder and ureters (18). There are several morphological common features between malignant BTs and high-grade transitional cell carcinoma of the urinary tract that may make challenging the differential diagnosis (19). Despite they share a transitional cell phenotype, there is considerable evidence that these two tumors represent distinct pathologic and clinical entities. Hull et al. identified criteria for mBT histological diagnosis consisting in the concomitant presence of both malignant and benign/borderline BT with clear stromal invasion by malignant epithelial components (20). BT/mBT are typically negative for WT1, ER and may have focal p53 positivity (15). Furthermore, BT have increased EGFR, Ras and Cyclin D1 expression with increasing degree of malignancy (21). Two studies performed sequencing and FISH analysis for several selected genes. None of their cases showed amplification of *EGFR*. However, they identified *PIK3CA, KRAS* and *MAP3K1* mutations, suggesting involvement of PI3-AKT and MAPK signaling (21–23). Although BT are almost exclusive ovarian neoplasms, they may constitute a highly unusual tumor of the testis (24). It remains a matter of debate whether ovarian-type epithelial tumors of the testis originate from the remnants of Müllerian ducts in the paratesticular connective tissue, epididymis and spermatic cord, or from Müllerian metaplasia of the mesothelium of the tunica vaginalis testis (11). To the best of our knowledge, only 7 cases of BT of the testis have been described to date with our patient being the eighth reported case (5–11). All cases are summarized in **Table 2**. The following features were identified: 1) age at onset range was 37-78 years, with majority of the patients aged >50 years; 2) a cystic mass, with testicular swelling was the most common clinical manifestation; 3) the size of the mass was variable; 4) diagnosis is based on pathological examination; 5) due to the rarity of reported cases, the survival rate is uncertain. This cancer is often misdiagnosed as hydrocele because symptoms are not specific, as well as laboratory results and imaging examinations. In our case, patient’s age, clinical presentation with synchronous liver metastases and negative tumor markers made difficult to hypothesize mBT. On histological examination, our case presented all morphological features of mBT, even if an epididymal localization has never been reported in literature. A crucial point in our case was represented by the differential diagnosis between BT and urothelial carcinoma. Usually, BT present a marked positivity for CK7 and are CK20 negative. Conversely, urothelial carcinomas have opposite IHC features with CK7 negative and CK20 positive. In our case, IHC profile confirmed BT diagnosis, excluding metastasis from urothelial carcinoma (**Figure 1**). Furthermore, TTF-1, PSA and CDx2 negative IHC excluded epididymal metastases from lung, thyroid, prostate and gastrointestinal tumors, respectively. Interestingly, our case was p63 low-positive as expected for mBT. In fact, p63 positivity is a diagnostic feature in benign BT, whereas mBT are typically p63 negative or slightly positive. Due to the rarity of epididymal mBT diagnosis, a further biopsy was needed in order to understand the nature of liver lesions. Histological examination of liver metastasis evidenced the same morphological and immunohistochemical features of primary lesion. Consistently, IHC negative for CK20, CDx2, TTF-1, CD10 and Vimentin excluded urothelial, gastrointestinal, lung, thyroid, kidney cancer and sarcoma metastasis. Based on the features of liver specimen, Pathologist concluded for glandular/transitional carcinoma diagnosis. Notably, a further evaluation by a second Pathologist was essential for liver metastasis final report. Our patient, due to liver and renal impairment died four months after diagnosis without systemic treatment. Generally, based on the available studies on ovarian BT, survival depends on the stage of the tumor (10). Consistently, following surgery the prognosis of benign BT is good, while the prognosis of mBT is poor. The 5-year survival of stage III/IV disease is ~0%, with only 1 known case surviving for >2 years after intensive systemic chemotherapy (25). Interestingly, among the seven cases of testicular BT reported in literature, survival information is available only for three of them with a favorable outcome after surgery (5, 7, 10). However, none of those patients had metastatic disease evidencing the essential curative role of primary surgery. Our patient was the first metastatic mBT described to date and his outcome was very poor, thus suggesting the same survival trend seen in ovarian mBT for stage IV disease. There is no information about therapeutic approach in these very rare cases; in fact none among the 7 published patients had received chemotherapy. Therefore, in absence of therapeutic standards, we decided to perform mutational analysis to find potential targets for treatment. Interestingly, Fibroblasts Growth Factor Receptor-3 (*FGFR3*) missense mutation (Y375C) and *PIK3CA* missense mutation (H1047R) were detected on primary BT. Surprisingly, the same *FGFR3* but not *PIK3CA* point mutation was found in mBT liver metastasis. Both *FGFR3* and *PIK3CA* mutations are reported as pathogenic in ClinVar database. In particular, *FGFR3* Y375C lies within the transmembrane domain of the Fgfr3 protein conferring a gain of function (26). A hypothetical structural model of mutant FGFR3 TM domain created using Swiss-Pdb Viewer Thv4.0.1. was built by Bodoor et al. In this model Y375C mutation results in the formation of a putative disulfide bridge with the corresponding cysteine at the opposite helix. The oncogenic ability of this mutation is also supported by a study in which a bladder carcinoma cell line expressing FGFR3b–Y375C lost its transforming ability upon treatment with an FGFR kinase inhibitor and/or FGFR3 shRNA (27). In Urothelial Carcinoma, Y375C is the second most common mutation with a frequency of around 22% (28). Although *FGFR3* mutations have also been found in multiple myeloma and carcinoma of the cervix, their frequency in these malignancies is much lower than in urothelial cancers (29, 30). Furthermore, except for benign skin tumors, other solid epithelial tumors have been found negative for *FGFR3* mutation, pointing to a specific role in bladder carcinogenesis (31). FGFR alterations represent a driver in urothelial cancers as well as a therapeutic target. Consistently, studies have been conducted to develop targeted and personalized therapies based on FGFR status. Several clinical trials are currently ongoing to assess the clinical efficacy of this class of multi-targeted TKIs, and to evaluate the potential implication of FGFR alterations as a biomarker (32). Recently, the FDA approved the oral multitarget Tyrosine-Kinase inhibitor erdafitinib for patients with metastatic bladder cancer harboring *FGFR2* or *FGFR3* alterations in progression after platinum-containing chemotherapy (33). Our mutational analysis shows FGFR3 mutation both on epididymal BT and liver metastasis, while PIK3CA mutation was only present in the primary tumor. Based on this concept, a personalized therapeutic approach with FGFR inhibitors could have represented a putative strategy for our patient. Unfortunately, the worsening clinical condition and disease progression led the patient to death in few months without treatment.

**Table 2:** Overview on Testicular/paratesticular and epididymal BT published in literature.

Authors, year(Ref)	Age (years)	ClinicalPresentation	Location	Size (cm)	Histology	Treatment	Survival (months)
Caccamo et al. (9)	62	NR	T and E	NR	BT	NR	NR
Goldman (7)	41	Intermittent aching sensation and tender mass	Left T	2.7x2.2x2.0	BT	S	36 (alive)
Nogales et al., (8)	37	Cystic mass	T	3	Mixed BTadenomatoid tumor	S	NR
Ross et al. (6)	61	Diabetes, cardiovascular complications	Paratesticular	0,6	BT	NR	NR
Vechinski et al. (5)	67	Inguinal hernia, swelling of the scrotum	T	7.3	BT	S	96 (dead for a second tumor)
Quan et al. (10)	55	Swelling and headiness of right T	T	6,5 x 4 x 3,5	BT	S	29 (alive)
Jones et al. (11)	60	Scrotal infection	T	0,7	BT	S	NR
Parcesepe et al., 2021	78	Left T mass	Left T and E	2,5	mBT	S	4 (dead)

NR, not-reported; T, testis; E. epididymis; BT, Brenner Tumor; mBT, malignant Brenner Tumor; S, Surgery (orchiectomy).

## Conclusion

To our knowledge, this study represents the first report of epididymal mBT with synchronous metastases in which mutational analysis has been performed. The rarity of testicular/epididymal BT does not provide high level of evidence for specific treatment. Actually, the majority of patients receive surgical resection in case of early stage disease. Therefore, a precision medicine-based approach could be a key in the management of rare cases. Our molecular findings should be further investigated and confirmed in larger series in order to better understand the role of FGFR-pathway in these tumors. In the light of our results *FGFR* mutations could represent a driver of malignant BT and a hypothetic therapeutic target.

## Data Availability Statement

The raw data supporting the conclusions of this article will be made available by the authors, without undue reservation.

## Ethics Statement

The studies involving human participants were reviewed and approved by The Ethics Committee “LAZIO 1” n. 2001/2017 prot. n. 2198. The patients/participants provided their written informed consent to participate in this study. Written informed consent was obtained from the individual(s) for the publication of any potentially identifiable images or data included in this article.

## Author Contributions

GG and PP conceived the study, performed the literature research, wrote the paper and assessed figure and tables. LC and AR collected the pathological samples and clinical data. LC, EM, and AR reviewed and confirmed the histological diagnosis. MS performed bioinformatics analysis. GC, MD’A, ES, and AS performed the literature research and critically reviewed the paper. PP and GG supervised the project. All authors contributed to the article and approved the submitted version.

## Funding

The project was funded by ARC-Net Centre for applied research on cancer and SAMAR Laboratorio di Istopatologia di Roma.

## Conflict of Interest

The authors declare that the research was conducted in the absence of any commercial or financial relationships that could be construed as a potential conflict of interest.
